# Method for hydrophytic plant sample preparation for light and electron microscopy (studies on *Phragmites australis* Cav.)

**DOI:** 10.1016/j.mex.2018.09.009

**Published:** 2018-09-28

**Authors:** Grigoriy M. Fedorenko, Aleksei G. Fedorenko, Tatiana M. Minkina, Saglara S. Mandzhieva, Vishnu D. Rajput, Aleksandr V. Usatov, Svetlana N. Sushkova

**Affiliations:** a Federal Research Center the Southern Scientific Center of the Russian Academy of Sciences, 41, Chehova st., Rostov-on- Don, 344006, Russia; b Southern Federal University, 105, Bolshaya Sadovaya street, Rostov-on-Don, 344006, Russia

**Keywords:** Method for hydrophytic plant sample preparation for light and electron microscopy, Method of plant preparation, Transmission electron microscopy, Ultrastructural analysis, Contamination with heavy metal

## Abstract

Nowadays there are no well-established, standard methods in electron microscopy despite its 50-year history. An excessive variety of research objects prompt researchers to modify and improve methodological approaches to sample preparation. One of the difficult objects to study by electron microscopy is hydrophytic plants, for example, *Phragmites australis* Cav. Traditional approaches to fixation and sample preparation do not give satisfactory results due to the peculiarities in structure and physiology of hydrophytic plants. The purpose of present research is modification description of the widespread method developed for double fixation of hydrophytic plant tissue for transmission electron microscopy. Suggested approach takes into account the features of hydrophyte plants.

• The developed method allows improving the quality of plant samples by additional fixatives imbibition and removing of air bubbles from aerenchyma tissue using a vacuum.
• The new step of sample preparation consisting in the layer-by-layer sample mixing in a special inclined mixer is applied for the embedding media penetrate sufficiently into the sample tissue.
• The process of samples inclusion in polymeric resins is carried out in the flat-bottom capsules. Compare to standard conical capsules, flat-bottom capsules allow strictly defined orientation sample pieces, that is permit to produce a semi-thin and ultra-thin slices of perpendicular to the longitudinal structures of the plant. This is especially important to conduct an adequate comparative analysis of dimensions, shape, and electron density of fragments and parts of the studying samples.

## Graphical abstract

**Figure:**
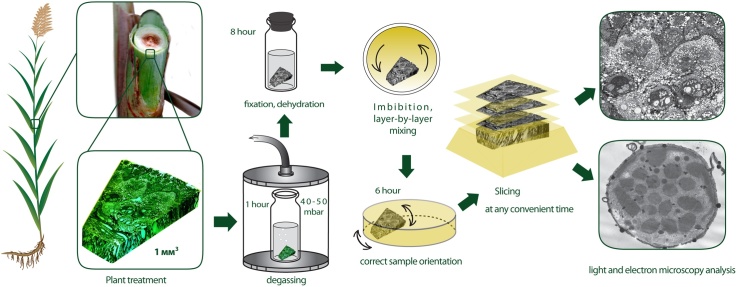

**Specifications Table**

**Table:** 

Subject area	*Agricultural and Biological Sciences*
More specific subject area	*Morphology, cytology, plant ultrastructure*
Method name	Method for hydrophytic plant sample preparation for light and electron microscopy.
Name and reference of original method	1 E.P. Guskov, G.M. Fedorenko, T.P. Shkurat, Ultrastructure of cells of the meristem of wheat in norm and after hyperbaric oxygenation, Cytology 27(1) (1985) 94–97. 2 A.V. Usatov, G.M. Fedorenko, L.B. Shcherbakova, E.V. Mashkina, Ultrastructure of the chloroplast of mustard *Brassica Juncea* as a measure of salt resistance, Cytology, 46(12) (2004) 1035 - 1042. 3 V.V. Rassadina, A.V. Usatov, G.M. Fedorenko, N.G. Averina, Activity of the system for chlorophyll biosynthesis and structural and functional organization of chloroplasts in a plastome en:chlorina-5 sunflower mutant, Russian Journal of Plant Physiology 52(5) (2005) 606–615. 4 V. Lysenko, G. Fedorenko, A. Fedorenko, E. Kirichenko, A. Logvinov, T. Varduny, Targeting of organelles into vacuoles and ultrastructure of flower petal epidermis of *Petunia Hybrida,* Revista Brasileira de Botanica 39(1) (2016) 327-336. 5 G. Gayer, Electronic histochemistry, Mir, Moscow, (1974), 235–236 [in Russian] 6 H.D. Coulter, Rapid and improved methods for embedding biological tissues in Epon 812 and Araldite 502, J. Ultrastruct. Res. 20, (1967) 346–355. 7 K.E. Wohlfarth-Bottermann, Die Kontrastierung tierischer Zellen und Gewebe im Rahmen ihrer elektronenmikroskopischen Untersuchung an ultradunnen schnitten, Naturwissenschaften 44 (1957) 287-288 8 T.P. O’Brien, N. Feder, M.E. McCully Polychromatic staining of plant cell walls by toluidine blue O, Protoplasma 59 (1964) 368-373 9 Reynolds E.S., The use of lead citrate at hight pH as an electronopaque stain in electron microscopy, J.Cell Biol. 17 (1963) 208–212
Reagents/tools	• 0.2 M phosphate buffer (pH 7.3) • 25% glutaraldehyde (SPI Supplies CAS#111-30-8) • 2% osmium tetroxide (SPI Supplies CAS # 20816-12-0) • Uranyacetate (SPI Supplies CAS # 6159-44-0) • 100% ethanol • 100% acetone • Epon812 embedding medium (SPI Supplies 02660-AB) • Methylene blue solution (SPI Supplies CAS # 7220-79-3 C.I. 52,015) • Copper mesh for TEM (SPI Supplies # 2010C-XA) • Glass vial • Flat-bottom capsules • Ultramicrotome (Leica EM UC6 or similar) • Transmission electron microscopy (Tecnai G2 Spirit BioTwin or similar) • Inclined mixer (where the vials with samples are positioned at an angle of 35° to the horizontal, which ensures a layered displacement of the embedding medium relative to the sample) • Thermo cabinet (temperature up to +62°C) • Desiccator with vacuum pump

## Method details

**Fig. 1:**
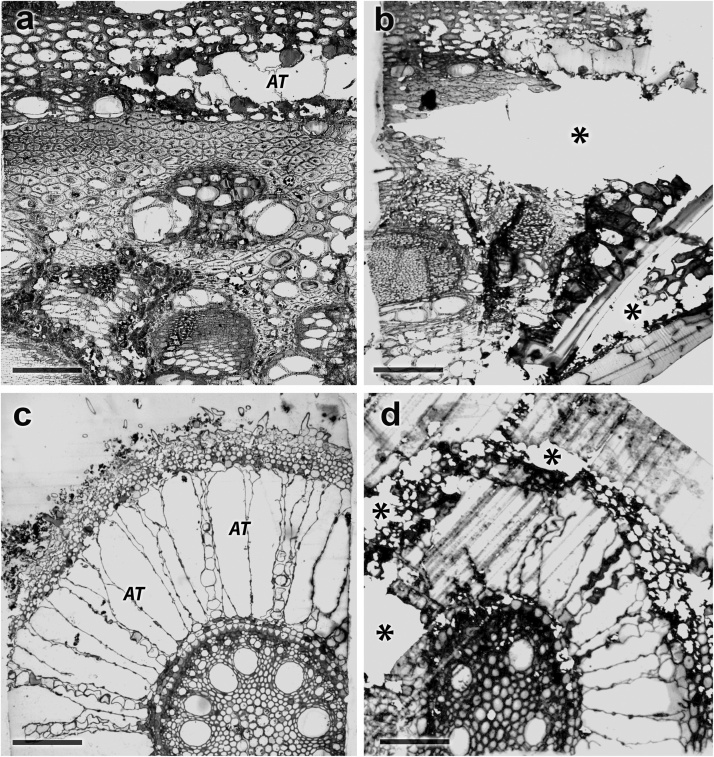
Semi-thin slices of *Phragmites australis* (а, b – stem, с, d – root) were produced by proposed method (a, c) and traditional method (b, d). AT - aerenchyma tissues, * - destructured tissues. The scale bar is (μk): a – 100, b – 100, c – 100, d – 100.

One of the major difficulties in hydrophyte plants sample preparation is the presence of extensive aerenchyma tissues in stems (to a lesser extent, leaves), rhizomes and roots. They contribute to the formation of air bubbles that prevent the penetration of fixatives solutions and embedding media into the plant tissue. This leads to the impossibility of producing the high-quality electron micrographs. The tissue samples poorly imbibed with fixing solutions and embedding medium are crumbled during the producing of semi-thin and ultra-thin slices (Fig. 1b, d). The presence of a massive cell wall in the hydrophyte plants tissues, which consists mainly of cellulose, hemicellulose, pectin and lignin, further prevents the fixatives and embedding media penetration into the sample.

The article describes a technique allows to overcome the above-mentioned problems by a sequential air bubble extraction and histological processing of sample tissues with fixing solutions by vacuum processing, and then imbibing with the embedding medium by layer-by-layer mixing (Fig. 1a,c, Supplementary data).

### Preliminary notes

Preparing of all necessary solutions, media and materials is required for a plant tissue processing and sampling. All sample processing procedures were performed at room temperature (23 ± 2 °C). Solutions and reagents stored in a refrigerator were brought to room temperature before using. The volume of solutions added to the glass vial with samples should be several times larger than the volume of the samples themselves, for example: for a sample of 1 mm^3^ the volume of the solution have to be no less than 5–6 ml. For convenience the whole process of fixation, dehydration and embedding can be divided into the three stages:

• Day one. Plant tissue sampling and fixing, partial dehydration; the samples are stored in the refrigerator overnight.
• Day two. Dehydration and placing in the embedding medium.
• Next three days. Polymerization of the prepared blocks with plant tissue samples. Then the samples can be stored without time limit.

### Plant treatment and fixation

The plants, *Phragmites australis* Cav., were sampled at the area of industrial sewage and slurry tanks near Kamensk-Shakhtinsky (Rostov Region, south of the Russian Federation).

A fragment was exsected from each plant sample and transferred to a drop of freshly prepared fixative placed on a hard surface. The fixative contains 1:9 mix of 25% glutaraldehyde (SPI Supplies CAS#111-30-8) and phosphate buffer (23 ml of 0,2 M NaH_2_PO_4_ + 77 ml 0,2 M Na_2_HPO_4_) [1]. The prepared fragment was cut into pieces so that when further embedded into blocks and sectioned, the cutting plane of the block was perpendicular to the longitudinal structures of the plant (conducting bundles, phloem and xylem elements, etc.). The sample was selected by size: at least one of its sides was less than 1 mm and the total volume not exceeding 1 mm^3^. Then, the samples were put to a small glass vial containing several milliliters of glutaraldehyde fixative. Vials with ajar lids were placed in a desiccator and air was slowly pumped out to reach a pressure of 40–50 mbar. The samples were then exposed to vacuum for one hour. After degasification and dropping to the bottom of the vial, the vacuum pump was turned off and slowly retained to atmospheric pressure level. The samples were kept in the same fixative medium for more than 3 h. Next step included samples washing in 0.2 M phosphate buffer (pH 7.3) and placing in 1% osmium tetroxide solution (1:1 mix of 2% osmium tetroxide (SPI Supplies CAS # 20816-12-0) in distilled water and phosphate buffer) for 2 h. Then the samples were dehydrated in the graded series of ethanol increasing concentrations (50%, 70%, 96% and 100%). The samples were stained with uranyl acetate (1% uranyacetate (SPI Supplies CAS # 6159-44-0) diluted in 70% ethanol) during their dehydratation with 70% ethanol [2].

*The method consists of the following step-by-step operations:*

• Place the samples in 2.5% buffer solution of glutaraldehyde (0.1 M, pH7.3).
• Keep the samples in the desiccator for 1 h to degasification process.
• Slowly increase the pressure in the desiccator to atmospheric and keep the samples in the fixative for 3 h.
• Remove the glutaraldehyde fixative and wash off the samples in the phosphate buffer solution (0.2 M, pH 7.3) two times for 20 min.
• Put the samples in a 1% osmium tetroxide solution (0.2 M, pH 7.3) for 2 h.
• Remove the osmium fixative, wash off the samples with 50% ethanol for 10 min.
• Put the samples in 1% uranyl acetate dissolved in 70% ethanol and refrigerate (+4 °C) overnight.
• Replace 1% uranyl acetate with 96% ethanol, then 100% ethanol (twice for 20 min), then 100% acetone.

### Imbibition in the embedding medium

**Fig. 2:**
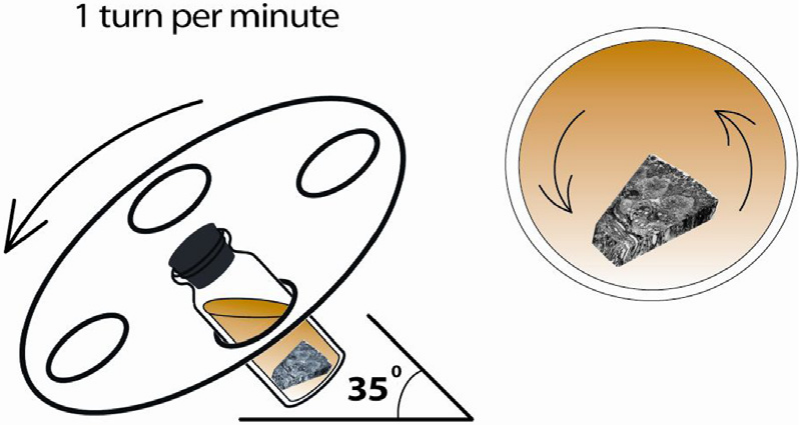
The layer-by-layer mixing in a special inclined mixer.

**Fig. 3:**
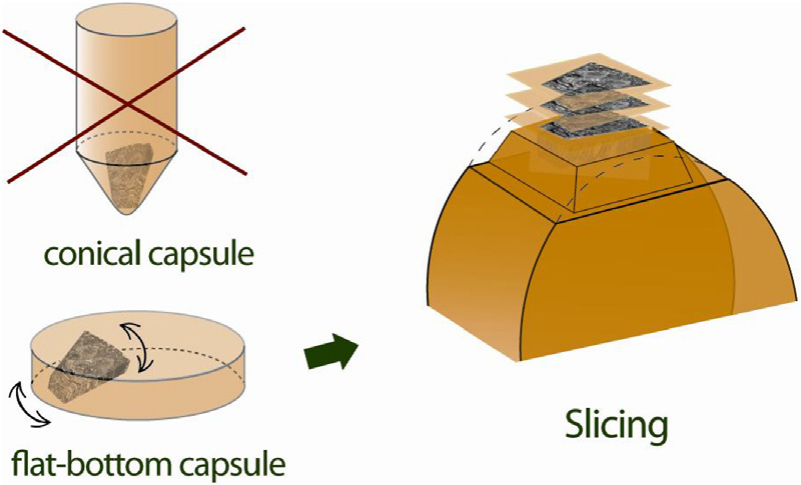
The strictly defined orientation sample in flat-bottom capsules compare to conical capsules.

Samples were placed into the Epon812 embedding medium (SPI Supplies 02660-AB) after dehydration [3]. Imbibition in the embedding medium is crucial, therefore, taking into account the peculiarities of the sample, it was used an inclined mixer. The vials with samples were positioned at an angle of 35° that ensures a layered displacement of the embedding medium relative to the sample (Fig. 2). Then the samples were placed into the flat-bottom capsules (Fig. 3).

It was important to obtain identical axial slices of control and experimental samples as extent as it possible in a comparative study of the *Phragmites australis*. The assuring of correct sample orientation in the block was required at the embedding stage. Unlike standard conical capsules, the flat-bottom capsules allow positioning a piece of the sample at the desired angle in the Epon block, which is very important for further sectioning. The polymerization stage included two steps. The first step was the sample polymerization at 37 °C for 24 h. During this time, there was possible to adjust the position of the sample because the embedding medium remained viscous enough. The second step was the final polymerization of embedding medium under the heating temperature in the oven up to 62 °C for 48 h.

*Make use of the following step-by-step embedding protocol:*

• Place the dehydrated samples in 2:1 (v/v) mixture of acetone: Epon (2 parts of acetone/1 part of the Epon). Place the vial into an inclined mixer for 120 min.
• Remove the mixture (2:1) and replace it with 1:1 (v/v) mixture of acetone: Epon (1 part of acetone/1 part of Epon). Place the vial into the inclined mixer for 90 min.
• Replace 1:1 mixture with 1:2 (v/v) mixture of acetone: Epon (1 part of acetone/2 parts of Epon). Place the vial into the inclined mixer for 90 min.
• Replace 1:2 mixture with 100% Epon. Place the vial into the inclined mixer for 60 min.
• Remove the Epon. Put the samples in the capsules prepared for embedding. Samples should be in the necessary position. Place the capsules into the oven with temperature 37 °C for 24 h.
• Check the samples position in the embedding medium. Adjust if necessary. Put it into the oven with temperature 62 °C for 48 h.

Sectioning of samples with subsequent light and electron microscopy analysis

• Extract the polymerized Epon blocks from the capsule using the scissors or a lancet.
• Turn the block on the milling cutter so that the front of the sample is parallel to the working face of the pyramid. Use a razor blade to form the pyramid shape.
• Fix the block in the sample holder and obtain semi-thin slices (700 nm) using an ultramicrotome (Leica EM UC6 or similar). Then transfer the slices to a drop of distilled water placed on a slide.
• Glue the slices to the glass by drying a drop of distilled water over a spirit lamp.
• Stain the slices by adding of 1% methylene blue solution drop (SPI Supplies CAS # 7220-79-3 C.I. 52015) [4].
• Heat the glass again over the spirit lamp. Do not allow the boiling of the dye solution.
• Rinse the slices in a stream of distilled water carefully. The slices are prepared to light microscope studying.
• Select the sample area of interest, correct the pyramid if necessary and produce ultra-thin (70–90 nm) slices.
• Transfer the slices to the copper mesh for TEM (SPI Supplies # 2010C-XA). They can be further contrast painted with lead citrate (1.33 g of lead nitrate + 1.76 g of sodium citrate + 30 ml of distilled water) for 1 min [5].

### Method validation

The advantage of the proposed method is the possibility of an ultrastructural comparative analysis of such complex objects for TEM, as root and stem tissue of hydrophytic plants. One of such plants was *Phragmites australis* that reflects the cumulative effects of environmental pollution from water and soil [6,7]. The aquatic macrophytes register heavy metal temporal fluctuations. All organs of *Phragmites australis* act as “bioindicators’’ and can be used as “biomonitors’’, defined as organisms providing quantitative assessment of the environmental quality [8]. The accumulating capacity of macrophytic plants is related to their physiological and anatomical features and the mechanism of heavy metal detoxification due to the predominant binding of metals by root cell walls [9,10].

**Fig. 4:**
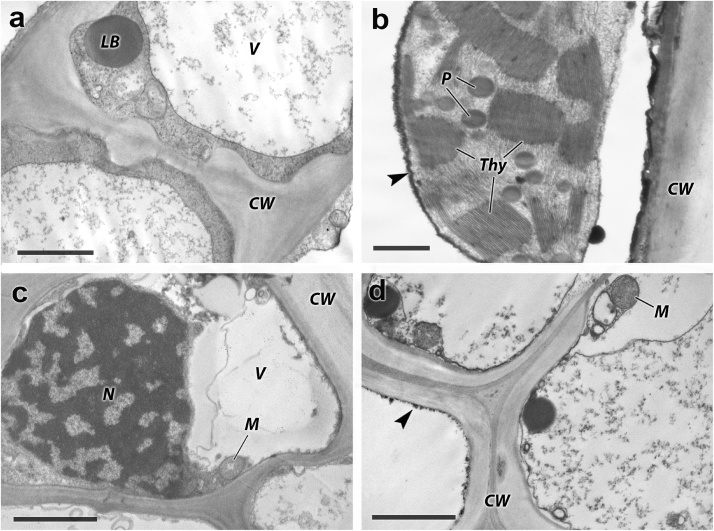
TEM micrographs of cross ultrathin slices of root (a, c) and stem (b, d) of *Phragmites australis* Cav., allowed identifying the cellular targets and the areas of the primary toxicity impact. CW – cell wall, LB - lipid body, N – nucleus, M – mitochondria, V – vacuole, P – plastoglobul, arrows – electron-dense sediments. The scale bar is (μk): a – 2, b – 0.5, c – 2, d – 2.

At the moment only few research articles presented the data on the ultrastructure of hydrophytic plants with the emphasis on leave tissue [11,12]. The results of investigation *Phragmites australis* Cav., grown under extremely high soil contamination, showed that the high quality of the obtained electron diffraction patterns allowed to detect changes in the ultrastructure of cell membranes as well as the main cytoplasmic organelles of root and stem (mitochondria, plastids, etc.) when exposed to a variety of environmental factors. The proposed method of preparing tissues for TEM can be used for all hydrophytic plants (Supplementary data). The use of TEM allowed identifying the cellular targets and the areas of the primary toxicity impact, determining structural mechanisms of changes in the level of absorption and translocation of nutrients from roots to sprouts, and their impact on the entire plant growth [[13], [14], [15], [16]] (Fig. 4).
