# Supplementary material for: Method for hydrophytic plant sample preparation for light and electron microscopy (studies on Phragmites australis Cav.)
Source: MethodsX. 2018 Sep 28;5:1213–20. doi: 10.1016/j.mex.2018.09.009 (PMC6190527; doi:10.1016/j.mex.2018.09.009)
Supplement: Supplementary file 1 [file mmc1.docx]

**Supplementary data**

The samples of *Oryza sativa* L., one of the cultivated agricultural hydrophytic plants, were prepared according to the suggested method. The quality of the samples preparation makes it possible to find subtle traces of the influence of unfavorable environmental factors (salinization) both at the light-optical and ultrastructural levels (Fig. 1-3).

The epibblem and pericyclic cell layer of the *Oryza sativa* cultivar “Boiarin” root, grown under conditions of salinity, thickened, and the mesoderm cells diminished in size and increased in quantity (Fig. a1). The epigram and pericycles of the *Oryza sativa* cultivar “Pokkali” root, grown in conditions of salinity, thickened insignificantly, the structure of the mesoderm changed little in comparison with the norm grown in conditions of salinity (Fig. b1). The epigram and pericyclic of the *Oryza sativa* cultivar “Ostap” root, grown in conditions of salinity, increased in size, compared with the norm, and the mesoderm changed the structure, preventing the radial movement of fluid in the root from the peripheral areas (Fig. c1).

**

**

**Fig. 1.** Microstructure of the central root of *Oryza sativa* (cross section) in normal and at 1.5% salinity. The scale bar is 100 μm. The arrows show the changes.

(a) The slice of the *Oryza sativa* cultivar “Boiarin” root, grown under normal conditions. (a1) The slice of the *Oryza sativa* cultivar “Boiarin” root, grown under conditions of salinity.

(b) The slice of the *Oryza sativa* cultivar “Pokkali” root, grown under normal conditions. (b1) The slice of the *Oryza sativa* cultivar “Pokkali” root, grown in conditions of salinity.

(c) The slice of the *Oryza sativa* cultivar “Ostap” root, grown under normal conditions. (с1) The slice of *Oryza sativa* cultivar “Ostap” root, grown in conditions of salinity.





**Fig. 2.** TEM micrographs of cross ultrathin slices of central root of *Oryza sativa* grown under normal conditions. The root epicle cells of the *Oryza sativa* cultivar “Boyarin” (a), “Pokkali” (b) and “Ostap” (c). B - vacuoles, M - mitochondria, arrow - cell wall. The root central cylinder cells of the *Oryza sativa* cultivar “Boyarin” (a1), “Pokkali” (b1) and “Ostap” (c1). N - nucleos. Scale bar: a - 0.5 μm, b - 1 μm, c - 0.5 μm, a1 - 1 μm, b1 - 1 μm, c1 - 0.5 μm.





**Fig. 3.** TEM micrographs of cross ultrathin slices of central root of *Oryza sativa* at 1.5% salinity.

The root epicle cells of *Oryza sativa* cultivar “Boyarin” (a), “Pokkali” (b) and “Ostap” (c). The root central cylinder cells of the *Oryza sativa* cultivar “Boyarin” (a1), “Pokkali” (b1) and “Ostap” (c1). Pg - plastoglobul, ER - endoplasmic reticulum. Scale bar: a - 1 μm, b - 1 μm, c - 0.5 μm, a1 - 0.5 μm, b1 - 1 μm, c1 - 1 μm.
